# Living Arrangement and Life Satisfaction in Older Malaysians: The Mediating Role of Social Support Function

**DOI:** 10.1371/journal.pone.0043125

**Published:** 2012-08-17

**Authors:** Hadi Kooshiar, Nurizan Yahaya, Tengku Aizan Hamid, Asnarulkhadi Abu Samah, Vajiheh Sedaghat Jou

**Affiliations:** 1 Department of Medical Surgical, School of Nursing and Midwifery, Mashhad University of Medical Sciences, Mashhad, Iran; 2 Department of Resource Management and Consumer Studies, University Putra Malaysia, Serdang, Malaysia; 3 Institute of Gerontology, University Putra Malaysia, Serdang, Malaysia; 4 Department of Social and Development Science, University Putra Malaysia, Serdang, Malaysia; 5 Department of Mathematics Education, Simon Fraser University, Vancouver, British Columbia, Canada; Cinvestav-Merida, Mexico

## Abstract

**Background:**

This cross-sectional and correlational survey examines the association between different types of living arrangements and life satisfaction in older Malaysians, while taking into account the mediating effects of social support function.

**Methodology and Findings:**

A total of 1880 of older adults were selected by multistage stratified sampling. Life satisfaction and social support were measured with the Philadelphia Geriatric Center Morale Scale and Medical Outcomes Study Social Support Survey. The result shows living with children as the commonest type of living arrangement for older adults in peninsular Malaysia. Compared to living alone, living only with a spouse especially and then co-residency with children were both associated with better life satisfaction (p<.01) and social support function (p<.01). The mediating effect of social support function enhanced the relation between living arrangements and life satisfaction.

**Conclusion:**

This study revealed that types of living arrangement directly, and indirectly through social support function, play an important role in predicting life satisfaction for older adults in Malaysia. This study makes remarkable contributions to the Convoy model in older Malaysians.

## Introduction

As in other countries, the number and proportion of older segments of the population affect Malaysia, and cause concern [1], [2]. The increasing numbers of older adults come with a challenge to maintain and promote life satisfaction. In addition, higher life satisfaction coupled with changing needs that may require social support, highlight the importance of understanding the living arrangements of older adults as a predictor of life satisfaction. Prior theories and evidence have demonstrated direct and indirect associations between types of living arrangements and the life satisfaction of older adults [3]–[5].

Social support is consist of interpersonal communication and interaction, love and understanding, caring and concern, affection and companionship, financial assistance, and respect and acceptance [6], [7].While, life satisfaction has been defined as “an internal and subjective perception, the individuals’ evaluation of their lives” [8]. Many studies have confirmed the contributions of social supports to the life satisfaction of older people [9]–[12]. However, some controversies exist about the exact nature or the characteristics of this relations. Several studies have shown the positive effects of social support, which enhanced life satisfaction [13]–[15]. Other studies showed that life satisfaction was not essentially enhanced by interpersonal interactions [16].

However, researchers from Western countries and the United States contributed most of the life satisfaction studies. Scientific reports of such investigations among Malaysian older adults are few; the associations among living arrangements, the social support function, and life satisfaction have not been investigated extensively. Studies about these concepts, specifically about life satisfaction, in one country may not be suitable for resolving the problem in another country. People in different living arrangements also vary across indicators of social support. To the extent that particular types of living arrangements define these social conditions, it is important to understand how they influence life satisfaction. Therefore, this study had two aims: 1) To examine the associations among four types of living arrangements and life satisfaction and 2) to examine the mediating effect of the social support function in the associations between types of living arrangements and life satisfaction.

### Fundamental Theory

The Convoy model of social relationships was considered the basis for this study because it determines relationships among social networks (living arrangements), social support, and life satisfaction based on research objectives. Kahn and Antonucci introduced the Convey model of social support in 1980 [17]. The model represents a theory for understanding social networks and social supports across the life course. The basic and fundamental principle of the Convey theory assumes social support as a crucial predictor of individual well-being [18]. The Convoy model of social support argues that social support comes from a relatively stable personal network of family, friends, and others [19]. Early research utilizing the Convoy model actually focused on older people [20], [21].

**Figure 1:**
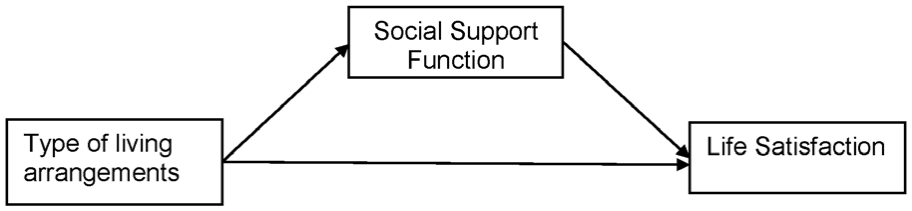
Conceptual framework mediating role of social support function on relation between type of living arrangements and life satisfaction.

Figure 1 shows the conceptual framework of this study based on the Convoy model previously described. According to this framework, one can expect relationships among types of living arrangements, social support function, and life satisfaction. In addition, types of living arrangements may have direct or indirect influences on life satisfaction through social support function.

## Methods

The Medical ethics committee from University Putra Malaysia approved the study and the waiver of written consent. The waiver of informed consent according to current guidelines on Good Clinical Practice and the Declaration of Helsinki was attained from University of Putra Malaysia Medical Ethics Committee [22]. For the present study, before starting the interviews, the consent forms were offered to all respondents. Respondents were informed that any data acquired via the interviews would be kept confidential and only the certified personnel would have access to the data and answering the questions was optional for them.

The study used a secondary database. The original database was collected using a cross-sectional and co-relational survey titled “Patterns of Social Relationships and Psychological Well-being among Older People in Peninsular Malaysia (PSRPWO)” For detailed information about methodology of this survey read paper that has been published earlier [22]. The survey divided Peninsular Malaysia into four zones to determine the locations of the study. Samples consisted of Malaysians 60 years and older who lived in the community. The total number of respondents included in this study was 1880 older adults. Respondents were selected by using a multistage stratified sampling (three stages) based on place of residence (rural, urban), age, and sex. The sample was representative of the geographical population of older adults in Peninsular Malaysia. Researchers used the direct interview technique as the method of data gathering. The mean of the interview time was about 30 minutes.

### Statistical Methods

In this study, data analysis used SPSS (version 13). Descriptive analysis was conducted for socio-demographic variables, Philadelphia Geriatric Center Morale Scale (PGCMS), and Medical Outcomes Study Social Support Survey (MOS-SSS) scales. The respondents’ mean scores on life satisfaction and social support across living arrangements were also compared using one-way analysis of variance (ANOVA).

Four steps of the Baron and Kenny approach [23] were used to establish the mediating effects of social support on the relationships among types of living arrangement and life satisfaction. In the first step, a correlation of the independent variable (types of living arrangements) to the dependent variable (life satisfaction) is required in path c′ (direct effect). For second step, a correlation of the independent variable to the mediating variable (social support function) is required in (path a). Third, both the independent variable and the mediating variable must be correlated to the dependent variable in path (ab) (indirect effect). Fourth, for complete mediation, the independent variable no longer effects on dependent variable after mediating variable has been controlled and path c′ is zero coefficients, however, for partial mediation, the absolute size of path c’ is reduced but is still different from zero when the mediator is introduced. Then with using AMOS 17.0 package, Structural Equation Model (SEM) with maximum likelihood carried out to evaluate and test mediation effects. SEM is a preferable data analysis strategy for mediation models [23]. The fit of the model to the data was assessed using chi square statistic, the Comparative Fit Index (CFI), Normed Fit Index (NFI), the non-normed Fit Index (NNFI), and the Root Mean Square Error of Approximation (RMSEA). Models with fit indices of >.95 and an RMSEA of <.06 indicate a close fit between the model and the data [24].

Exploratory data analysis conducted on the variables in this study revealed that these variables were normally distributed. In order to assess normality, the analysis used “skewness” and “kurtosis” between ±1. The sample size was large so “variance ratios” were used to measure homogeneity of variance between variables. These tests were conducted to ascertain the normality and homogeneity of variances, which were assumptions of the parametric tests for this study.

### Measurement

In this study, socio-demographic variables included ethnicity (Malay = 1 and non-Malay = 0), sex (male = 1 and female = 0), marital status (married  = 1 and non-married = 0), level of education (formal school = 1 and non-formal school = 0), age, personal income, and household income. The (PGCMS) was used as a measure of life satisfaction, following many previous studies [25]–[27]. The (PGCMS) consists of 17 mostly dichotomous items, with scores of 1 for each “yes” response and 0 for each “no” response. The total score ranges from 0–17, with a higher score indicating a higher level of life satisfaction. The (MOS-SSS) measured social support functions with 19 items measuring perceived social support. For each item, the respondents must indicate how often each support is available when they need it. For the MOS-SSS, each item has a Likert scale of 1 to 4: (1) none of the time, (2) some of the time, (3) most of the time, and (4) all the time. Choosing (4) is a full positive response and choosing (1) is a full negative response. To obtain a score for the MOS-SSS, responses are summed across all 19 items then transformed to a scale from 0–100. The range of total scores was from 19 to 76. A higher score indicates better function of social support [28]. Types of living arrangements were living with others, living with children, living only with spouse, and living alone.

### Validity and Reliability of PGCMS and MOS-SSS

Exploratory factor analysis evaluated the underlying construct validity of the MOS-SSS. The internal consistency of the (PGCMS) and (MOS-SSS) were evaluated using Cronbach’s coefficient alpha, and both instruments had high internal consistency and reliability. Internal consistency and reliability for these instruments were α = 0.76 and α = 0.95, respectively. The validity and reliability of the (MOS-SSS) was previously evaluated in an American adult population [29]. Chai et al. (2009) examined the reliability and factorial structure of the (PGCMS) on this data set. Their results showed the overall reliability of the scale as proposed by Lawton [30].

## Results

The average and standard deviation of respondents’ age were 69.79 and 7.36, respectively, with a median age of 69 years, and a range from 60 to 112 years. A majority (76%) of the respondents were in a young-old age group (between 60 and 74), and only 3.7% of respondents were in an oldest-old group (>84). About 75.60% of the respondents identified themselves as “Malay and other Bumiputera,” while 24.40% stated “non-Malay” (Chinese Malay and Indian Malay). Female and male were 52.61% and 47.39% of the respondents respectively. Approximately, 56% of the respondents were married at the time of the interview. About, 37% of respondents had never attended formal school. The mean of personal income and household income were 564.09 (SD = 616.43), and 1398.36 (SD = 1383.55) Ringgit of Malaysia respectively.

The mean and standard deviation of life satisfaction scores were 11.49 and 3.5, respectively, with a range from 0 to 17. Considering a comparison with responses in a study involving 928 respondents, a score ranging from 13 to 17 would be high life satisfaction, 10 to 12 would be a mid-range, and less than 9 would be at the low end of the scale [31]. According to this cutoff point, about 75% of respondents in this study were towards the upper limit of life satisfaction. The mean score of the social support function was 64.05. Table 1 summarizes the various types of living arrangements among older adults.

**Table 1:** Frequency distribution of respondents by type of living arrangements.

Type of living arrangements	Frequency	%
Living alone	175	9.31
Living with spouse only	155	15.53
Living with children	1341	71.33
Living with others	72	3.83
Total	1880	100

The Pearson statistical test showed there was significant correlation between social support and life satisfaction (r = .36 p<0.001). One-way ANOVAs also revealed that there were significant differences in life satisfaction across types of living arrangements and social support function scores. The Tamhane post-hoc comparisons of the groups indicated that the social support and life satisfaction scores of older adults who lived alone were lower than the scores of respondents in other types of living arrangements. There were no significant differences in social support scores between “living with children” and “living only with spouse” (Table 2).

**Table 2:** Mean of life satisfaction, and social support scores by type of living arrangements.

	Type of living arrangements (%)				
Variables	living alone	living only with spouse	living with children	living with other	Test
Life Satisfaction	9.58 (3.80)_a_	12.22 (3.09)_b_	11.65(3.42)_c_	9.96(3.57)_a_	F^1^ = 25.14**
Social Support	46.51(23.39)_a_	66.67(23.17)_b_	66.23(20.94)_b_	55.51(26.19)_c_	F^1^ = 41.02**

Note. Means with sharing a row subscript (a, b, c) are not statistically different according to the Tamhane procedure, Standard deviations appear in parentheses bellow means. 1 = Welch’s F.

** p<.01.

### Mediating Effect

**Figure 2:**
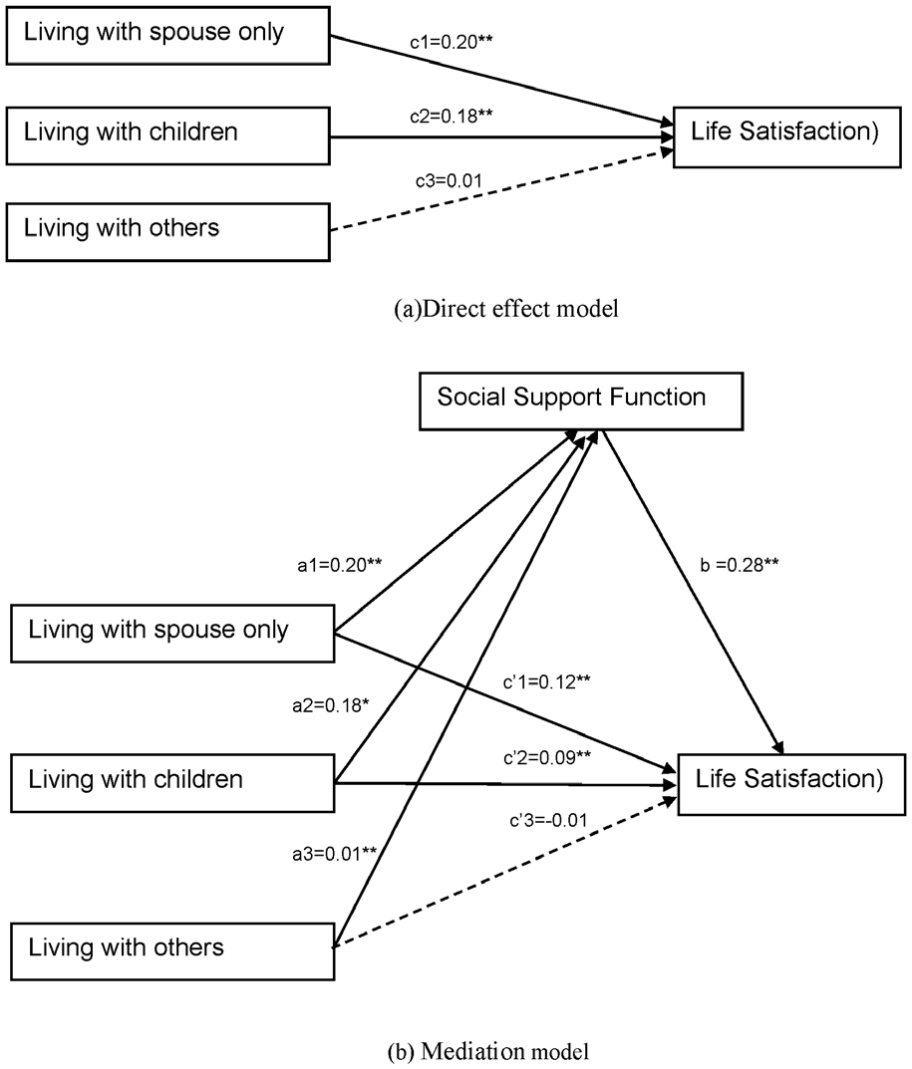
Direct effect model (a) of types of living arrangements and life satisfaction, and mediation model (b) including social support function as mediator between types of living arrangements and life satisfaction.

First tested was a direct effect model of type of living arrangements and life satisfaction (Total effect). After removing non-significant relationship path between “living with others” and “life satisfaction” the model provided good model fit for the data χ^2^ (1, N = 1880) = 0.60, p = .44, CFI of 1, NFI = 1, NNFI = 1, and an RMSEA value smaller than.05. When compared to living alone, the total effects of two other types of living arrangements had a significant effect on life satisfaction: living with spouse only (β = 0.20, p<0.01), and living with children (β = 0.18, p<0.01) (Figure 2-a).

Furthermore, this study examined the mediating effect of social support using Baron & Kenny’s causal steps’ strategy [23]. The mediation model provided a very good fit for the observed data which indicated by the non-significant chi-square: χ^2^ (1, N = 1880) = 0.04, p = .84; CFI of 1.00, NFI = 1, NNFI = 1, and an RMSEA value smaller than.05. There was a significant relationship between the social support function and life satisfaction (β = 0.28, p<0.01). Living with spouse had the strongest effects on the social support function (β = 0.20, p<0.01) compared to living with children (β = 0.18, p<0.01) and living with others (β = 0.01, p<0.01). According to Baron and Kenny’s criteria, the direct effects of two types of living arrangement on life satisfaction (c_1_′ = 0.12, c_2_′ = 0.09) were smaller than the total effect (c_1_ = 0.20, c_2_ = 0.18); therefore, this model confirmed the partially mediation of the social support function (Figure 2-b).

## Discussion

This study has focused on examining the association between the types of living arrangements and life satisfaction in older Malaysians, taking into account the mediating effects of social supports. The findings of this study are in agreement with other studies that confirmed the relationship between living arrangements and psychological well-being [4], [32], [33]. Types of living arrangements are important to the life satisfaction of older adults because living arrangements act as a powerful function in defining social roles and providing social support functions and interaction [34].

The results of this study showed that older adults who live alone have lower life satisfaction than do those who live in other types of living arrangements. These findings are supported by Yah (2004) Borg (2006), and Shin, (2012) who found a well-known association between living alone and lower life satisfaction [35]–[37]. In agreement with this study Agrawal (2012) shows that elderly who are living alone have poorer health status, than elderly who are living with their family [38]. However, contrary to this study, Ng et al. (2004), Chou (2006), and Brajkovi (2012), reported no significant differences regarding psychological well-being between older adults who lived with their families and those who lived alone [5], [], [39,40].

The results of this study showed that respondents who lived with a spouse only or lived with children had higher life satisfaction. Several prior studies found that subjective well-being was higher for those who lived with family members (spouse or children) than older adults who lived alone [41]–[43]. In line with this study, Cong and Silverstein (2004) found that living in a three-generation household was most beneficial to older parents’ psychological well-being [44]. Chan (2005) found the older adults who live alone or live with non-relatives are in a worse condition than those who live with one adult child [45]. In the 2005, a UN report, “Living arrangements of older people around the world” noted that, “today as in the past, co-residence with older and younger kin is an important element in the system of intra-family support transfers, which affects the well-being of older and younger individuals” [46].

The living arrangements of older populations also have an influence on the demand for formal and informal support systems [47]. The results of this study showed that the lowest level of social support was available for older adults who lived alone, compared with other types of living arrangements. Beard et al. (2001) concurred with the present study, as it found that support relationships are slightly varied in different types of living arrangements [48]. The results of this study are consistent with Ng, Phillips, and Lee’s (2002) findings, which reported the highest social support for older adults who lived with their children. They also confirmed higher life satisfaction for older adults who lived with a spouse or children compared to those who lived alone [49]. Gow et al. (2007) found the largest association between the amount of support received from the spouse and life satisfaction [50]; this is in agreement with the current study. Russell (2007) reported that older adults who live alone were largely restricted from access to other social supports [4]. The spouse could be an important source of support, compared to other types of living arrangements [51]. Lower levels of social support in people who live alone may be for this reason that they do not have a “close-knit” network within the household [52].

The results of this study demonstrated that social support related to life satisfaction. Similar results have also been found in other studies [53]–[55]. Bowung, Farquha, and Browne, (1991) found a slight association among the availability of family, interaction, and subjective well-being. Social support contributions to the life satisfaction of older people are rather mixed and the exact nature or characteristics of social relations are controversial issues [10]. The results of this study are consistent with the findings of several studies that have shown the positive effects of social support, including the enhancement of life satisfaction [13], [15], [56]. On the other hand, contrary to the findings of this study, Mancini, Quinn, Gavigan, and Franklin (1980) showed that life satisfaction was not essentially enhanced by interpersonal interactions [16]. A negative relationship between life satisfaction and social interaction was observed by Lowenstein and Katz (2005); they concluded that social interactions increased distress among older people [57]. An unsupportive and unfriendly relationship can be destructive, leading to poor social interactions, distress, and disappointment, all of which affect well-being [58].

Collectively, previous theory and evidence suggest that living arrangements can affect psychological well-being through characteristics associated with relationships in the household [52]. Waite and Hughes (1999) reported poor well-being patterns among individuals living in the least supportive, and most demanding, living arrangements [34]. Types of living compositions other than living with a spouse or with children had well-being disadvantages due to the lack of stability and security in close social relationships. Under conditions of low social support, where the balance of positive to negative interactions within the home favors the latter, it is clearly better to live alone than to live with a spouse or family member [4]. The results showed that living in a three-generation household was most beneficial to older parents’ psychological well-being. The findings of the current study are in accordance with Cong et al. (2004) who found that receiving financial and emotional support from children reduced the negativity affecting older adults [44].

### Conclusion

The findings of this study showed that living with children was the most common type of living arrangement for older adults in peninsular Malaysia, as it is in the majority of countries in East Asia. Co-residency with children, and living specifically with a spouse, was associated with better life satisfaction compared to living alone; some important part of this life satisfaction is due to the indirect effect of the social support function. This study makes several important contributions to the Convoy model of social relationships in older Malaysians. The findings of this study revealed that living arrangements play an important role in the life satisfaction of adults in Malaysia, in both direct and indirect ways through the social support function. One can specifically assume that social support is a crucial predictor of life satisfaction.

### Limitations

The data set for this study was from a cross-sectional study. Hence, data were collected at one point in time; a longitudinal research design would be more precise to measure phenomena that changed over time. In this study, instruments of social support function measured only available support and there was some limitation in measuring received support and reciprocity of support between older adult. Therefore, a single perspective limits the present study. The other parties’ assessments of the relationships were not available. This study could not measure some of the variables in the “Convoy model,” as Kahn and Antonucci (1980) proposed [17], because the secondary data used in this study were impossible to measure. They proposed measuring social networks by showing three concentric circles to respondents, and having them determine their network members in these three circles according to their importance. However, in this study, living arrangement measured network members.

### Implication

The current study will be of benefit to gerontologist, psychologist, social workers, business professionals, religious leaders, elderly people and those intimately associated with them, in understanding of some factors that are considered to be key role players in life satisfaction and the mechanism that type of living arrangements play an important role to predict life satisfaction in older adults in Malaysia.
